# A four-year analysis of the predictors of overnight stay in day-case laparoscopic cholecystectomy and the prevention strategies within two centers in the UK: a double-centric retrospective observational study

**DOI:** 10.1186/s12893-026-03867-3

**Published:** 2026-07-13

**Authors:** Mohamed Abuahmed, Rahi Karmarkar, Muhammad Naeem, Rahel Rashid, George Eskandar, Merene Varghese, Mayank Mishra, Jeremy Wilson, Conor Magee

**Affiliations:** 1 The University of Edinburgh, Edinburgh, S12 3DF UK; 2 The Royal Wolverhampton NHS Trust, Wolverhampton, UK; 3 Liverpool University Hospitals NHT trust, Liverpool, UK; 4 Wirral University Teaching Hospitals NHS Trust, Wirral, UK

**Keywords:** Laparoscopic cholecystectomy, Same-day, Day-case, Overnight stay, Unexpected admission

## Abstract

**Background:**

Laparoscopic cholecystectomy (LC) is the mainstay treatment of benign gallbladder disease, most commonly gallstones. Although the recent guidelines of British Association of Day-Surgery (BADS) and World Society of Emergency Surgery (WSES) recommend same-day discharge (i.e. day-case surgery), there is still a considerable proportion of cases that get admitted unexpectedly overnight. The primary aim of this study is to determine the predictors of overnight stay and consequently develop prevention strategies.

**Materials and methods:**

This was a retrospective study involving 913 patients who underwent intended day-case LC between 2020 and end of 2023, in two centers in the UK; Wirral University Teaching Hospitals (WUTH) and the Royal Wolverhampton (RWT) NHS Trusts using electronic data records. We evaluated the predictors of overnight stay among demographics, indication of surgery, as well as the predictors of conversion to open surgery, which absolutely resulted in unexpected admission.

**Results:**

Nine hundred and thirteen patients were included in this study. Increased age, female gender and anesthesia ASA III grade patients were found to be predictors of unexpected admission (*p* < 0.001). In addition, the presence of acute cholecystitis and CBD stones as indications for surgery predicted overnight stay as well (*p* < 0.001). Moreover, age and the presence of choledocholithiasis/cholangitis (post ERCP/ passes out stones) were associated with conversion to open surgery, which resulted in 100% overnight stay (*p* = 0.013, *p* = 0.004, respectively), where older patients and those who had CBD stones had 1.05 times and 6.79 times higher odds of conversion, respectively. Furthermore, increased age and male gender were more likely to have a drain inserted (*p* = 0.004, *p* < 0.001, respectively).

**Conclusion:**

Although many of the predictors of overnight stay insertion were non-modifiable risks like age, gender and ASA grade, strictly following the guidelines concerning the management of postoperative symptoms like pain, nausea and vomiting as well as the prioritisation of day-cases in the operative list may play a role in avoiding overnight stay. Nevertheless, these factors did not significantly alter the outcomes in our study.

## Introduction and background

Gallstone disease, especially the symptomatic one, is one of the most common problems in the current surgical practice, affecting mainly females in their third decade of age, who are usually either overweight (BMI 25–29 kg/m^2^) or obese (BMI more than 30 kg/m^2^) [1]. The mainstay of treatment is cholecystectomy, through the laparoscopic minimally invasive approach, which has become the gold standard of care based on the latest guidelines in the UK [2]. Nowadays, it is recommended to perform laparoscopic cholecystectomy as a day-case, where patients get discharged on the same day as a standard of care. It is recommended that the target is to perform 75% of LC as an ambulatory surgery in each centre in the UK [3], which has been shown by multiple high quality studies to be a safe and feasible option for patients undergoing LC [4]. Many studies have addressed the criteria for discharge after day-case LC, most importantly are patient factors such as: age below 65, absent significant co-morbidities, ASA grade I/II, absence of prior abdominal surgery or duct stones, in addition to operative factors such as elective surgery [4]. Patients with acute cholecystitis, on the other hand, usually require urgent early LC based on the current guidelines, and those patients are more susceptible to overnight stay [5]. However, there has been attempts to increase the rate of same-day discharge even for those patients [2].

Nevertheless, proper discharge planning (DP) before day-surgery to determine whether a patient is more likely to be discharged or admitted after surgery forms an essential part of the patient care, as well as plays a pivotal role in the resource allocation in over-pressurised health care systems [6]. Unexpected overnight stay for planned day-case surgery puts a huge burden on healthcare systems through increased costs, longer length of stay, and inability to accommodate more urgent patients [7].

Multiple studies have shown the cost-effectiveness of standardization of DP processes and have, therefore, recommended the usage of AI models and machine learning to predict the time and destination of discharge as well as length of hospital stay (LOS) based on age, gender, ethnicity, admission type, rural/urban residency and chronic conditions [6].

The primary aim of our study is to offer further insights into the predictors of overnight stay after day-case LC through studying a large cohort of patients over a considerable period of time, in two centres in the UK with different ethnic backgrounds, which is turn is intended to broaden the understanding and further development of DP of such patients, with better resources allocation. 

## Methods

This was a retrospective observational study using electronic data records used at NHS foundation trusts in the UK; Clinical Web Portal [Internet]. Edinburgh (UK): NHS Lothian, and Cerner Millennium [computer software]. Kansas City (MO): Cerner Corporation.

This study was conducted at two district general hospitals in the UK; Wirral University Teaching Hospitals (WUTH) and The Royal Wolverhampton NHS trust (RWT), over a period of four years, between January 2020 and October 2024. Data collection period was started on December 2024 and ended in August 2025, and follow up period was 30 days after surgery to assess readmission rates. This study was approved by the local clinical governance committee in both NHS trusts.

All adult patients, aged 18 years and older, who were admitted to hospital with the intent of performing a day-case LC, within the intended time period were included in this study. Follow up was checked by reviewing electronic data records. Excluded were children (< 18 years old), and patients with no intention for same day discharge. The study included 913 patients, no gender predilection, and they were representative of multiple ethnic groups (more white British among the WUTH patients, and more Asian among the RWT patients). Ethnicity of patients was determined by the electronic records. This sample size offered a statistical power of 90%, with a margin of error of 0.35%. A 95% confidence interval was used to ensure the precision of our estimates. Patients were then divided into the overnight stay group and same-day discharge group.

Primary outcome was to identify predictors of overnight stay based on demographics and surgery indications using statistical analysis. Secondary outcomes were predictors of conversion to open surgery, comparing the timing of start of surgery, and operative duration and their effect on social issues and overnight admission, identifying the effect of the management of postoperative pain, nausea and vomiting (PONV) in preventing unexpected admission, predictors of drain insertion, unexpected admission as a predictor for readmission and postoperative complications and finally the prediction of overnight stay in hot gallbladder patients. Potential confounders were the absence of local policy regarding the routine prescription of postoperative analgesia and antiemetics by anaesthetists in the recovery room in one of the centres (RWT).

Outcomes were extracted through reviewing an individual record for each patient, no report linking was done. There was an adequate identified follow up period, which is 30-days after surgery, and no patients were lost for follow-up.

Quantitative variables like age, BMI, LOS and operative duration, in addition to qualitative variable such as gender, ASA grade, indication for surgery, conversion to open surgery, timing of surgery start, causes of overnight stay and 30-day complications and mortality, prescribing postoperative symptomatic treatment and documentation of clear postoperative plan were all extracted from electronic data records mentioned above.

### Statistical analysis

All statistical analyses were performed using Jamovi (version 2.6), an open-source statistical software (The Jamovi project, 2025, https://www.jamovi.org). Continuous variables such as age, BMI and operative duration were expressed as mean ± standard deviation (SD) and compared using the independent t-test or Mann–Whitney U test as appropriate. Categorical variables such as gender, ASA grade, indication for surgery, conversion to open surgery, timing of surgery start, causes of overnight stay and 30-day complications and mortality were expressed in frequencies and percentages, and were compared using the Chi-square or Fisher’s exact test.

Variables found to be significant in univariate analysis (*p* < 0.05) were entered into a binary logistic regression model to identify independent predictors of overnight stay, and conversion to open surgery. In addition, the effect of timing of surgery start, operative duration and the management postoperative pain and nausea/vomiting (PONV) on overnight admission was also calculated using the same method. Moreover, another univariate analysis was done for prediction of drain insertion, which in turn led to unexpected admission. Lastly, the predictors of hot gallbladder among demographic factors, operative start time and duration were calculated using univariate analysis as well. Model fit was evaluated using the Omnibus test of model coefficients and Nagelkerke R. Predictive performance was assessed using the classification accuracy and the area under the receiver operating characteristic (ROC) curve (AUC). Statistical significance was set at *p* < 0.05.

All figures and ROC curves were generated using Jamovi software (version 2.6). The use of ELM (Edinburgh access to Language Models), based on the GPT-4 architecture, provided by the University of Edinburgh, in this manuscript was limited to language optimisation, grammatical modifications and was not use to generate any new material in this manuscript.

## Results

### Demographics

In our study, 913 patients who had an intended day-case laparoscopic cholecystectomy were included. Females represented the majority of cases with 685 patients (75%) (F: M = 3:1), with a median age of 55 (IQR 24), and a mean BMI 35.9 (+/- 8.89), and an ASA score ranging from I-III, where two thirds of the patients (609 patients, 66.7%) had ASA II score, 177 patients and 125 patients had ASA I and ASA III scores, respectively.

Patients were then divided into two groups according to overnight admission or same-day discharge. The comparison in demographics and indications for surgery between both groups is demonstrated in Table 1.

**Table 1:** Comparison between same-day discharge and overnight stay patients according to demographics and indications for surgery

Variable	Same-day discharge (*n* = 570), n (%)	Overnight stay (*n* = 343), n (%)	*P* value
Age (median/IQR)	54 (24)	55 (24)	< 0.001 t=-5.888
Gender (Female) (%)	451 (78.9%)	238 (69.38%)	< 0.001 χ²=13.6
BMI (mean, Std)	39.6 (+/-14.55)	38.79 (+/-14.04)	0.464 T = 0.733
ASA I(%)	137 (23.99%)	40 (11.66%)	< 0.001 χ²=41.7
ASA II (%)	381 (66.72%)	228 (66.18%)	
ASA III (%)	52 (9.10%)	73 (21.28%)	
Biliary colic (%)	478 (83.71%)	256 (74.63%)	0.002 χ²=11
Acute cholecystitis (%)	69 (12.08%)	78 (22.74%)	< 0.001 χ²=17.9
Gallstone pancreatitis	26 (4.55%)	20 (5.83%)	0.301 χ²=2.4
CBD stones/Cholangitis	25 (4.37%)	37 (10.78%)	< 0.001 χ²=13.9
GB empyema	2 (0.35%)	2 (0.58%)	0.607 χ²=0.265
GB polyp	12 (2.10%)	5 (1.45%)	0.48 χ²=0.49
Porcelain GB	1 (0.17%)	0%	0.438 χ²=0.602
Miscellaneous	4 (0.70%)	4 (1.16%)	< 0.001 χ²=105

*IQR* Interquartile ranger, *BMI* Body mass index, *Std* Standard deviation, *ASA* American society of Anesthiologists, *CBD* Common bile duct, *GB* Gallbladder

Miscellaneous indications for laparoscopic cholecystectomy included mucocele (three patients), adenomyosis (three patients), gallbladder cancer (one patient diagnosed by MDCT), cholesterosis (one patient).

### Univariate analysis to predict overnight stay based on demographics and surgery indications

As per our two-centric study, both age and female gender were predictors of unexpected admission after day-case laparoscopic cholecystectomy; (t=-5.888, 95% CI -8.37 to -4.19, *p* < 0.001) and ( χ²=13.6, 95% CI -0.169 to -0.0495, *p* < 0.001), respectively. In addition, more overnight stay patients had ASA III grade than discharged patients, and this difference was significant (χ²=41.7, *p* < 0.001). This means that lower ASA grade is one of the determinants of discharge after day-case surgery.

Furthermore, more day-cases were indicated as a result of biliary colic and chronic cholecystitis (*p* = 0.002, χ²=11, 95% CI -0.0901 to -0.145), while more overnight stay patients had their surgery due to acute cholecystitis (*p* < 0.001, χ²=17.9, 95% CI 0.0545 to 0.158), as well as common bile duct (CBD) stones and cholangitis (*p* < 0.001, χ²=13.9, 95% CI 0.0271 to 0.101), and these differences were statistically significant.

### Predictors of conversion to open surgery

The conversion to open surgery during LC lead to unexpected admission, longer hospitalisation, and more 30-day complications including surgical site infections, abdominal incisional hernias and readmission. In our study, only 14 patients (1.53%) had open conversion of laparoscopic cholecystectomy. This was accompanied by 100% unexpected admission, with longer operative duration (159 min +/- 79.1 min) in comparison to (79.3 min +/-35.8 min) in non-converted cases, which was statistically significant (*p* < 0.001, U = 1803, 95% CI -114 to -32).

**Fig. 1:**
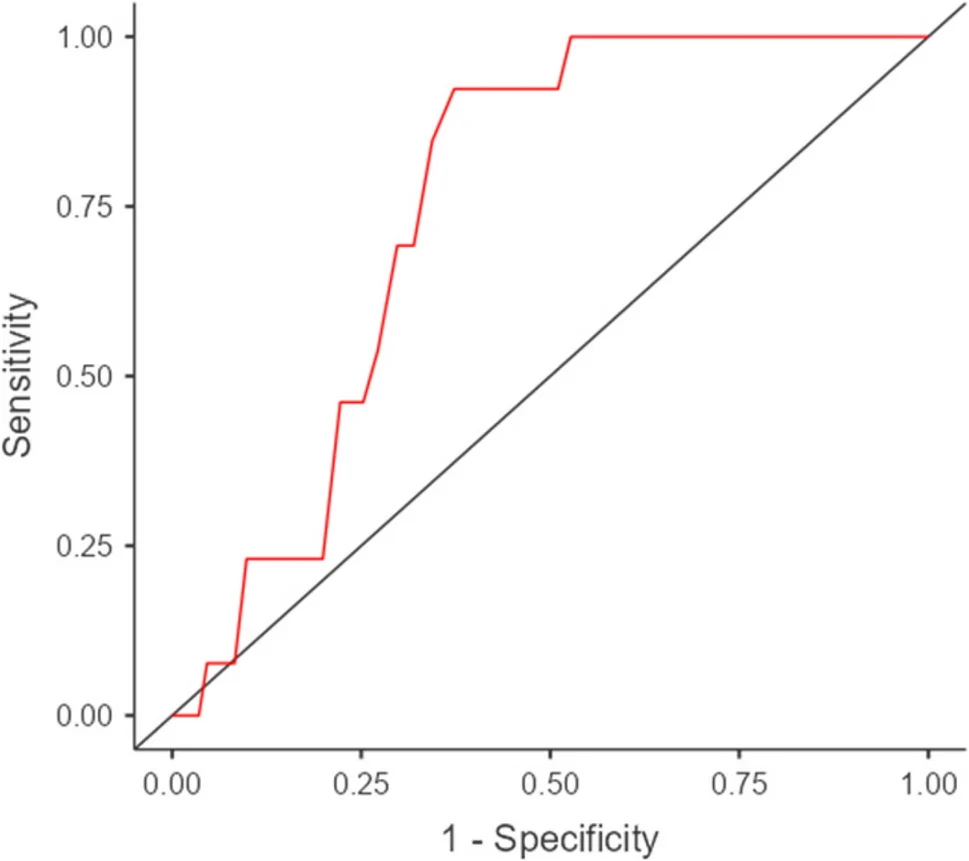
ROC Curve demonstrating the prediction of age to conversion to open surgery

**Fig. 2:**
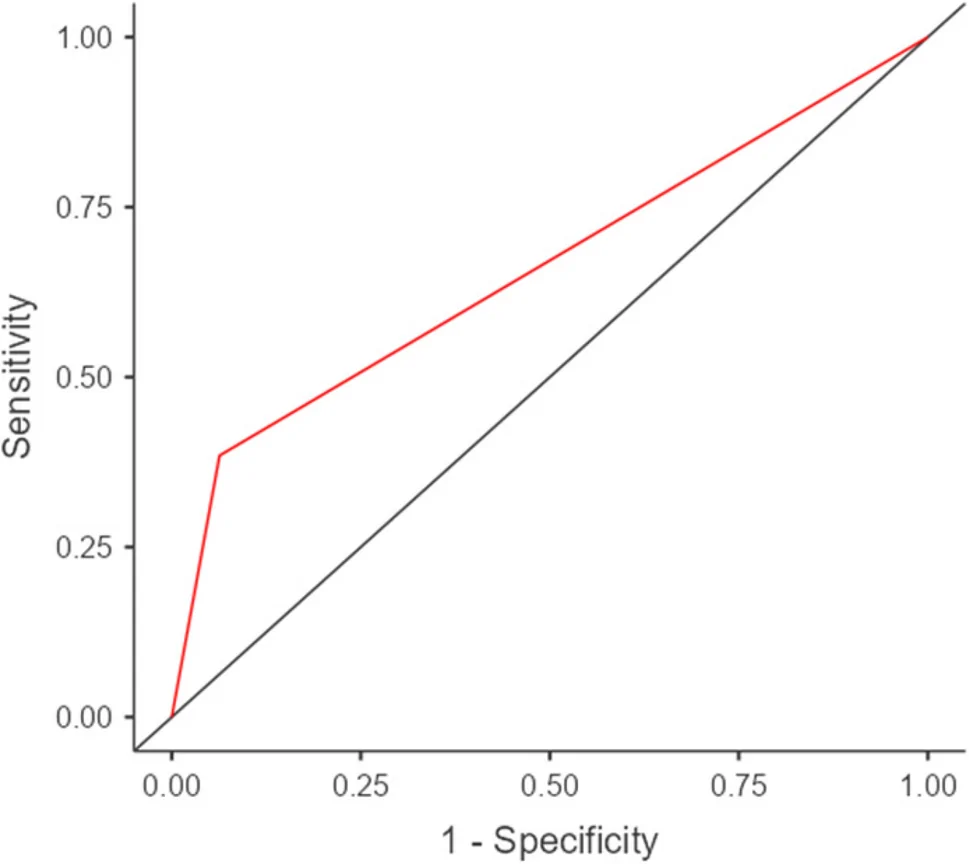
ROC Curve demonstrating the prediction of Choledocholithiasis/cholangitis to conversion to open surgery

On univariate analysis, increased age and choledocholithiasis/cholangitis (post ERCP patients or patients who passed out CBD stones) were associated with conversion to open surgery and were entered into the logistic regression model. The overall model was statistically significant (Omnibus test of model coefficients, χ² = 6.19, *p* = 0.013, χ² = 8.16, *p* = 0.004, respectively), indicating that the predictors as a group reliably distinguished between converted and non-converted cases. The model explained 13.7% of the variance in conversion status (Nagelkerke R² = 0.137). Older patients had 1.05 times higher odds of conversion (OR 1.05, 95% CI 0.092–0.0217, *p* = 0.022), and those who had CBD stones had 6.79 times higher odds of conversion (OR 6.79, 95% CI 3.1036–0.6065, *p* = 0.002). The model demonstrated good discrimination with an area under the ROC curve (AUC) of 0.793. The ROC curve for the logistic regression model is shown in Figs. 1 and 2, illustrating the model’s discrimination between conversion and non-conversion cases (AUC = 0.793).

Lastly, conversion to open surgery was associated with 6.33 times more 30-day readmissions than non-converted cases (OR = 6.33, χ² =9.56, 95% CI 1.67-24, *p* = 0.002), where three patients were readmitted within 30 days, two of them were due to surgical site infection.

### Unexpected overnight stay

This subgroup of patients comprised more than one third of patients (343 patients, 37.5%). The reasons behind their stay are shown in Table 2.

**Table 2:** Cases of unexpected admissions with frequences

Cause of unexpected admission	Number of patients *n* (%)
Postoperative pain	98 (28.57%)
PONV	50 (14.57%)
Postoperative cardiovascular issues	18 (5.24%)
Intraoperative drain insertion	113 (32.9%)
Postoperative urinary retention	32 (9.32%)
Intraoperative bleeding	15 (4.37%)
Social issues	28 (8.16%)
Increase O2 requirements	19 (5.53%)
Intravenous antibiotics requirement	14 (4.08%)
Difficult operation	19 (5.53%)
Drowsiness	8 (2.33%)
Consultant recommendations for monitoring	21 (6.12%)
Miscellaneous	7 (2.04%)

*PONV* Postoperative nausea and vomiting

### Timing of start of surgery, and operative duration and their effect on social issues and overnight admission

In our study, performing a univariate analysis showed that the patients whose operations started in the afternoon (i.e. after midday) were 1.06 more likely to be socially admitted than those who had their operations in the morning, but this difference was not statistically significant (OR 1.06, χ²=0.0235, 95% CI 0.478 to 2.37, *p* = 0.878).

However, for operative duration, results showed that patients who were socially admitted had even lower mean operative duration (82.2 +/-28.3) in comparison to non-socially admitted patients (103 +/- 47.7), and this difference was in fact statistically significant (U = 3071, 95% CI 2–30, *p =* 0.026). These results were in contrast to the mainstream thinking that timing of surgery start and the duration of operation might increase the possibility of overnight stay due to social issues related to lacking of a responsible adult to accompany patients after their discharge based on the NHS policy.

### The management postoperative pain and nausea/vomiting (PONV) for the prevention of overnight admission

In our study, the management of postoperative symptoms like pain, nausea and vomiting did not improve the rates of unwanted admission following day-case laparoscopic cholecystectomy, where the cases that had severe symptoms, required more analgesia and antiemetics, but warranted overnight stay as well; 151 patients among the admitted patients (44%) had PO management of their symptoms in comparison to 29 patients (5.08%) among the discharged patients, which means that patients who has PO management of their symptoms where 28.4 times more likely to be admitted (OR 28.4, χ²=199, 95% CI 16.8 to 48, *p* < 0.001). The reason behind this controversy is the lack of routine prescription of analgesia and antiemetics during the postoperative recovery, and they are only required when the symptoms are severe and warrant admission anyways.

### Predictors of drain insertion and in turn overnight admission

Our study shows that 113 patients had drain insertion (12.37%), all of which were unexpectedly admitted overnight (100%) for drain monitoring and management of pain associated with drain and to aim for discharge the following day after drain removal. Univariate analysis of drain insertion showed that increase age was significantly associated with drain insertion, where the mean age among the patients with drain was 60.2 +/-13.7 in comparison to 55.1 +/-16.6 among no drain patients (t=-2.902, 95% CI -8.58 to -1.65, *p* = 0.004). In addition, male gender was associated with significantly more drain insertion than females (χ²= 16.5, 95% CI 0.247 to 0.620, *p* < 0.001). Moreover, a higher BMI was associated with an increased number of drain insertion (40.4 +/-21.4 in comparison to 39.7 +/-13.4), however, this difference was not significant (*p* = 0.730).

**Fig. 3:**
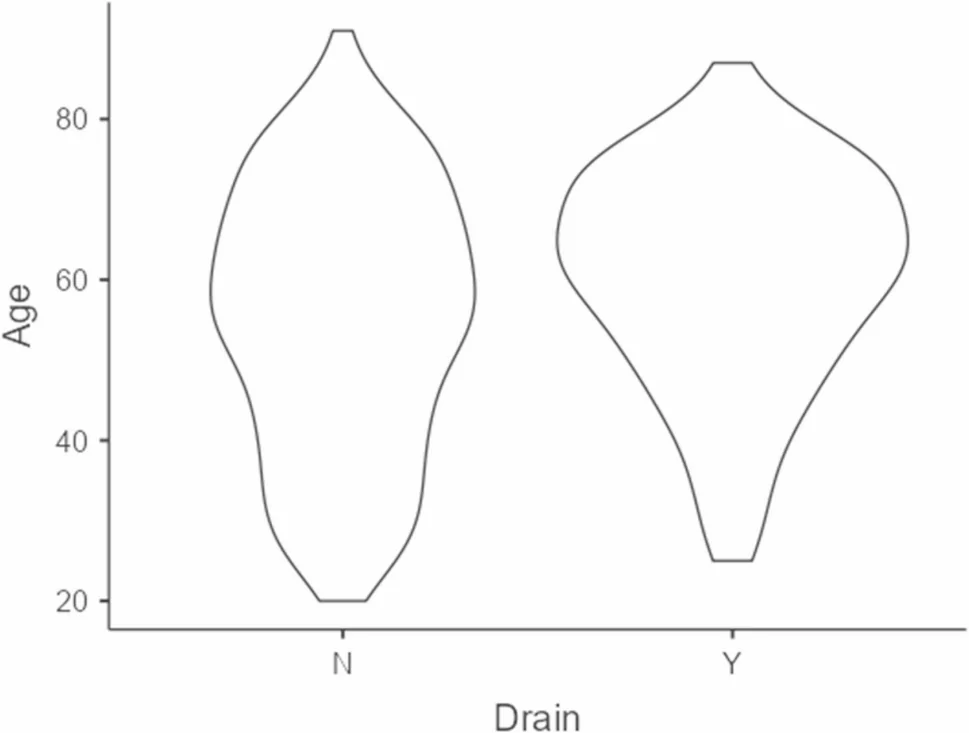
Plot that shows the relationship between age and drain insertion

**Fig. 4:**
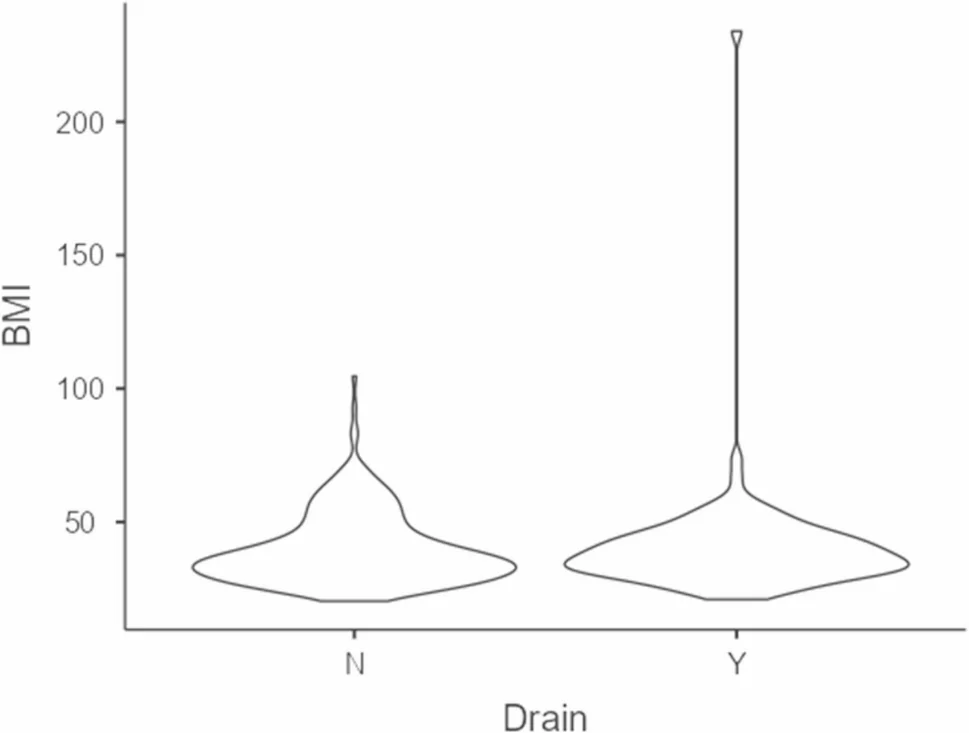
Plot that shows the relationship between BMI and drain insertion

**Fig. 5:**
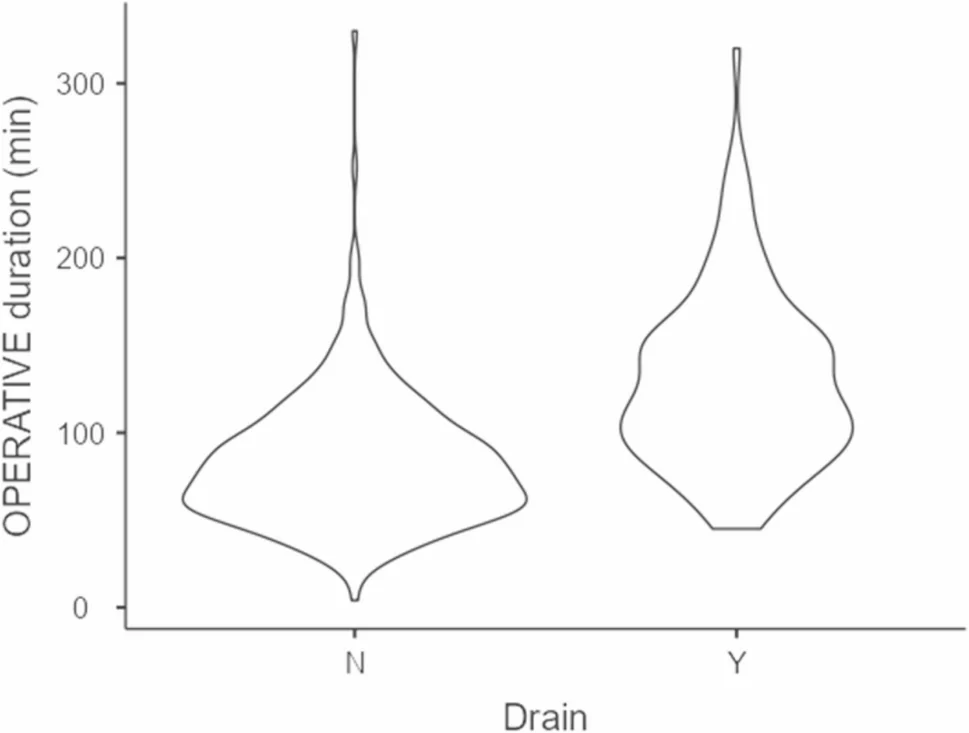
Plot that shows the relationship between operative duration (min) and drain insertion

Different ASA grades, on the other hand, showed no significant differences in drain insertion among patients (*p* = 0.541). Moreover, longer operative duration was associated with drain insertion, likely due to complexity of the operation, where the mean duration was 129 +/-49.4 min in drain inserted cases, in comparison to 83.2 +/-37.6 min in no drain cases (t=-9.960, 95% CI -54.88 to -36.78, *p* < 0.001). However, operative start time (morning vs. afternoon) did not show significant difference between drain and no drain cases (*p* = 0.710). This can be demonstrated by Figs. 3, 4 and 5.

**Fig. 6:**
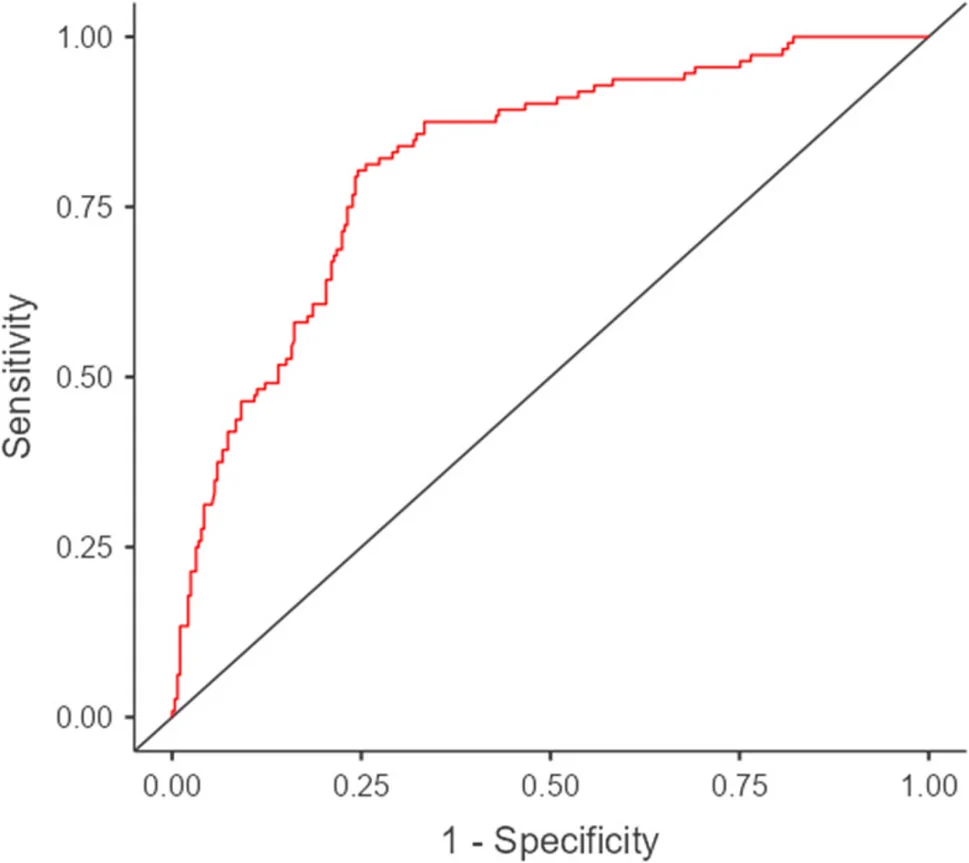
ROC Curve showing prediction of drain insertion by increased age, male gender and longer operative duration

Multivariate analysis showed that age, male gender and operative duration were predictors of drain insertion. The overall model was statistically significant (Omnibus test of model coefficients, χ² = 4.25, *p* = 0.039, χ² = 6.75, *p* = 0.0009, χ² = 75.09, *p* < 0.001, respectively), indicating that the predictors as a group reliably distinguished between drain and no drain cases. The model explained 30.7% of the variance in conversion status (Nagelkerke R² = 0.307). Older patients had 1.021 times higher odds of drain insertion (OR 1.021, 95% CI 1.0065 to 1.036, *p* = 0.004), and longer operative duration led to 1.0252 times higher odds of drain insertion (OR 1.0252, 95% CI 1.0187 to 1.0317, *p* < 0.001). The model demonstrated good discrimination with AUC of 0.817. The ROC curve for the logistic regression model is shown in Fig. 6.

**Fig. 7:**
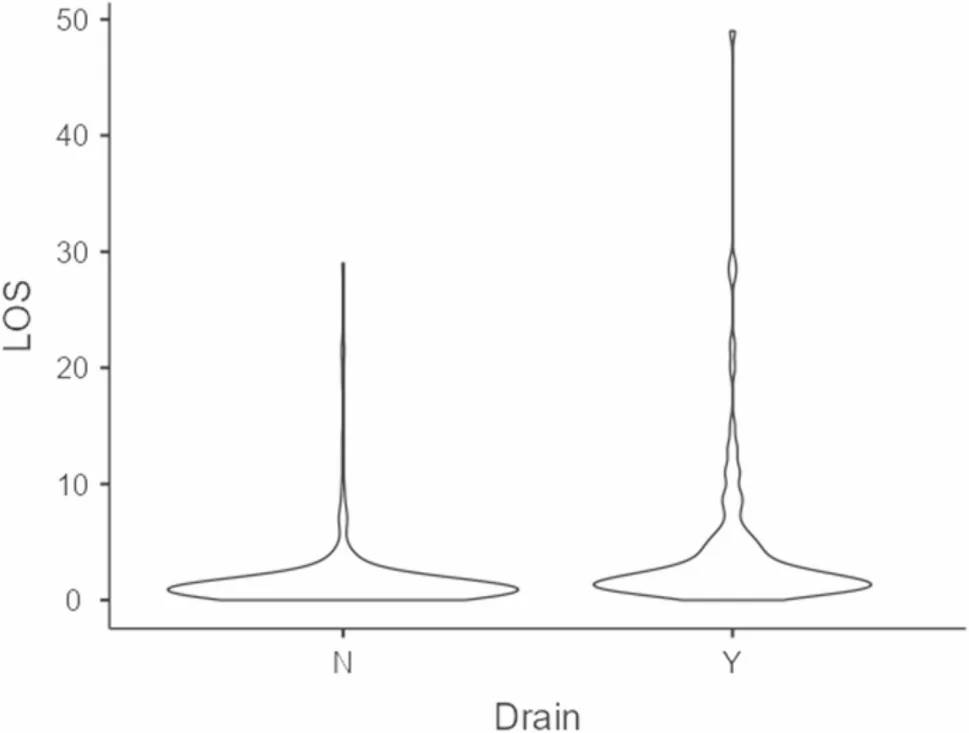
Plot that shows the relationship between drain insertion and LOS

Furthermore, drain insertion was associated with more length of stay, (LOS), where patients who has a drain inserted spent a mean of 4.17+/-6.61 days in comparison to 1.62+/-3.07 days, which was a significant difference, (t=-5.26, 95% CI -3.5 to -1.59. *p* < 0.001), as shown in Fig. 7. However, 30-day readmission and complications were almost the same between drain and no drain patients, (*p* = 0.969).

### 30-day complication rate and readmission

Out of 913 patients included in this study, 44 (4.81%) patients were readmitted within 30-days due to postoperative complications. The reasons and frequencies for the readmissions were shown in Table 3.

**Table 3:** Causes of readmission after laparoscopic cholecystectomy

Readmission	*N* (% from total number of patients)
Postoperative surgical site infection	13 (1.4%)
Postoperative bleeding	3 (0.32%)
Postoperative obstructive jaundice	8 (0.87%)
Postoperative bile leakage	2 (0.21%)
Miscellaneous causes	18 (1.86%)

Miscellaneous causes included: PONV, Pain, collection, hematemesis, acute pancreatitis, and stone passer

Out of these 44 patients, 24 patients were unexpectedly admitted after surgery, in comparison to 19 patients who were not, and these differences were statistically significant (χ² =5.68, OR = 2.07, 95% CI 1.13 to 3.8, *p* = 0.017). This means that the readmitted patients were 2.07 times more likely to be unexpectedly admitted after surgery. Moreover, most of the patients had clear documentation of postoperative plan except for 30 patients (3.28%). The absence of clear discharge plan did not lead to significant increase in readmission rate (*p* = 0.112).

### Hot gallbladder

**Fig. 8:**
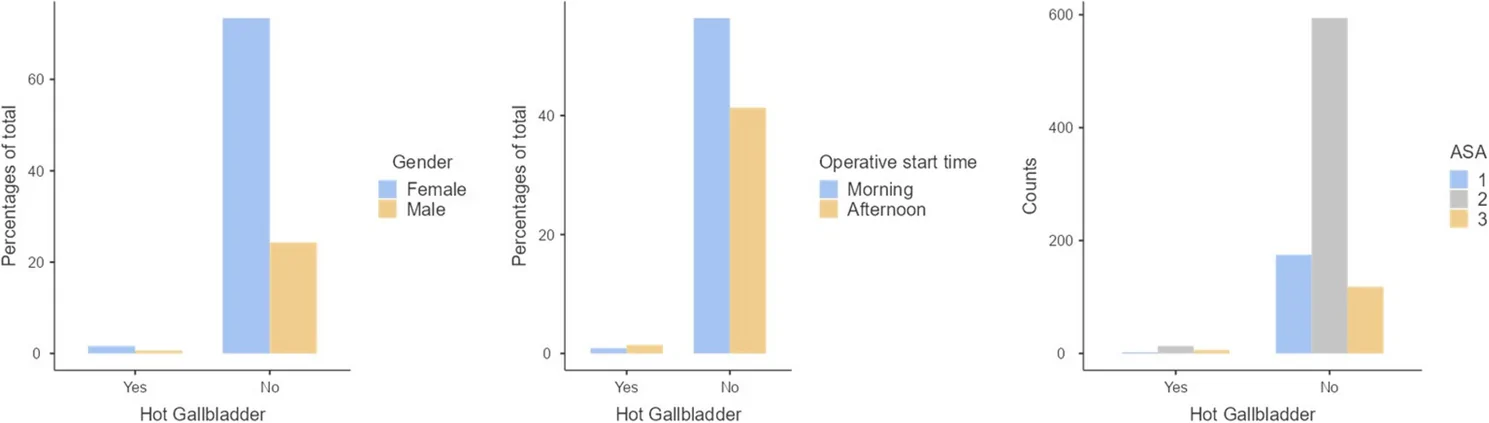
Distribution of hot gallbladder cases according to gender, operative start time and ASA grade

A subgroup analysis was done for hot gallbladder patients, although these patients usually undergo an emergency surgery, which means they are less likely to be a day case surgery. Our study included 21 patients who underwent hot gallbladder surgery, 15 patients (71.42%) were females, with median age of 53 years old (IQR 24) and mean BMI of 31.15 +/-6.69. 90% of the cases (19 patients) were ASA II and only 10% were ASA III. Only higher BMI (mean 32.94 +/-9.02) was associated with hot gallbladder (t=-1.93, 95% CI -5.98 to -3.1, *p* = 0.054). All of age (mean 52.67 +/-16.94 years), gender, ASA grade, LOS (1.10+/-2.83 days), operative start time (61.9% in the afternoon) and operative duration (mean 80.14 +/-50.77 min) were not significantly different among hot gallbladder cases, where most patients were discharged before 19:00 pm from the ambulatory unit (*p* = 0.898,0.698,0.096,0.956,0.073 and 0.959, respectively), as shown in Fig. 8.

Less than half of the hot gallbladder cases were admitted unexpectedly (9 patients, 42.8%) and 12 patients were on the other hand discharged, without no significant difference (*p* = 0.606). In addition, none of the patient underwent conversion to open surgery, while only one patient was readmitted within 30 days from surgery due to obstructive jaundice.

## Discussion

In our study, we aimed at identifying the predictors of overnight stay after day-case LC based on demographics, surgical indications, operative start time and duration, management of PONV and pain and the presence of hot gallbladder, where 343 patients underwent overnight stay after day-case LC, and each of increased age, female gender, ASA III, presence of acute cholecystitis, CBD stones were associated with increased unexpected admission after day-case surgery. Moreover, drain insertion and conversion to open surgery were associated with overnight stay. Among the 113 patients who had a drain inserted, male gender and age above 60 were the most predictors, while in case of the 14 patients whose surgery was converted to open, these were associated with increased age, longer operative duration and the presence of CBD stones. We also found that the readmitted patients were 2.07 times more likely to be unexpectedly admitted. Finally, it has been found that only higher BMI was associated with hot gallbladder surgery, which itself did not lead to significantly more overnight stay, complications or readmission.

Many studies have addressed various strategies aiming to promote same-day discharge. El-Sharkawy, et al. developed CAAD score (cholecystectomy as a day-case) based on age, gender, ASA grade, previous hospital admission, primary indication for surgery, preoperative investigations, and the presence of thick walled gallbladder or CBD stones and concluded that a CAAD score of ≤ 5 was associated with 80.8% successful day-case cholecystectomy [8]. In addition, the most recent guidelines of BADS recommend a detailed preoperative assessment in order to plan and predict unexpected overnight stay [2].

Regarding demographics, various retrospective studies have concluded that old age, above 60 years significantly lead to unexpected admission [9, 10], together with higher BMI, where studies have shown that a BMI of ≥ 50 kg/m^2^ had a 10% higher possibility of overnight stay, and increased ASA grade [11]. In addition, BADS recommended avoiding the routine use of drains because they are associated with infection, bleeding, and postoperative pain [3]. Concerning conversion to open surgery and its predictors, the CholeS audit showed that older age, female sex, the presence of CBD stones, higher ASA grade, thick-walled gallbladder and dilated common bile duct (CBD) diameter led to more conversion to open [12].

Although many studies did not find significant difference in the rate of readmission among patients who had day-surgery versus overnight stay, a large cohort study addressing the rates of day-surgery during COVID-19 pandemic in the UK found a significant reduction in readmission among the day-surgery patients [13]. Lastly, the hot gallbladder patients are more associated with pain and sepsis than the elective gallbladder surgery patients, which may lead to more overnight stay [2]. These findings were confirmed by multiple other studies addressing the overall financial benefits and safety of hot gallbladder surgery [14, 15].

Our study included a more than average sample size which offered a statistical power of 90%, with a margin of error of 0.35%, this enhanced the external validity and the generalisability of this study. One limitation to our study is the absence of clear data on the total costs inflicted by overnight stay patients after LC. Another limitation is the absence of local hospital policies regarding the postoperative symptoms management in one of the two centers (RWT).

## Conclusion

Increased age and ASA grade represent the greatest predictors of overnight stay, together with female gender, although male gender is more associated with drain insertion with lead to increased unexpected admission. The presence of CBD stones and acute cholecystitis lead to more hot gallbladders performed which usually lead to more hospital stay. Guidelines suggest managing postoperative symptoms like pain, nausea and vomiting as well as the prioritisation of day-cases in the operative list in order to avoiding overnight stay. Further studies are needed to develop machine learning models in order to predict overnight stay. 

## Data Availability

The datasets used and/or analysed during the current study are available from the corresponding author on reasonable request.
